# Reliability and measurement error of sensorimotor tests in patients with neck pain: a systematic review

**DOI:** 10.1186/s40945-023-00170-9

**Published:** 2023-08-15

**Authors:** Simone Elsig, Lara Allet, Caroline Henrice Germaine Bastiaenen, Rob de Bie, Roger Hilfiker

**Affiliations:** 1 School of Health Sciences, University of Applied Sciences and Arts Western Switzerland, HES-SO Valais-Wallis, Rathausstrasse 25, 3954, Valais Leukerbad, Switzerland; 2 Department of Epidemiology, Research Line Functioning, Participation and Rehabilitation, CAPHRI - Care and Public Health Research Institute, Maastricht University, PO Box 616, 6200 Maastricht, the Netherlands; 3 School of Health Sciences, University of Applied Sciences and Arts Western Switzerland, HES-SO Valais-Wallis, Chemin de l’Agasse 5, Valais Sion, Switzerland; 4 The Sense Innovation & Research Center, Sion and Lausanne, Switzerland; 5 Department of Medicine, Faculty of Medicine, University of Geneva, Geneva, Switzerland

**Keywords:** Neck pain, Sensorimotor tests, Reliability

## Abstract

**Background:**

Neck pain is one of the leading causes of years lived with disability, and approximately half of people with neck pain experience recurrent episodes. Deficits in the sensorimotor system can persist even after pain relief, which may contribute to the chronic course of neck pain in some patients. Evaluation of sensorimotor capacities in patients with neck pain is therefore important. No consensus exists on how sensorimotor capacities of the neck should be assessed in physiotherapy. The aims of this systematic review are: (a) to provide an overview of tests used in physiotherapy for assessment of sensorimotor capacities in patients with neck pain; and (b) to provide information about reliability and measurement error of these tests, to enable physiotherapists to select appropriate tests.

**Methods:**

Medline, CINAHL, Embase and PsycINFO databases were searched for studies reporting data on the reliability and/or measurement error of sensorimotor tests in patients with neck pain. The results for reliability and measurement error were compared against the criteria for good measurement properties. The quality of evidence was assessed according to the modified GRADE method proposed by the COSMIN group.

**Results:**

A total of 206 tests for assessment of sensorimotor capacities of the neck were identified and categorized into 18 groups of tests. The included tests did not cover all aspects of the sensorimotor system; tests for the sensory and motor components were identified, but not for the central integration component. Furthermore, no data were found on reliability or measurement error for some tests that are used in practice, such as movement control tests, which apply to the motor component. Approximately half of the tests showed good reliability, and 12 were rated as having good (+) reliability. However, tests that evaluated complex movements, which are more difficult to standardize, were less reliable. Measurement error could not be evaluated because the minimal clinically important change was not available for all tests.

**Conclusion:**

Overall, the quality of evidence is not yet high enough to enable clear recommendations about which tests to use to assess the sensorimotor capacities of the neck.

**Supplementary Information:**

The online version contains supplementary material available at 10.1186/s40945-023-00170-9.



## Introduction

Neck pain is the second most common musculoskeletal problem [1]. It is one of the leading causes of years lived with disability worldwide and represents an increasing burden on healthcare systems [2–4]. The economic burden of neck pain, in terms of treatment costs, lost productivity and work-related problems is high [1]. The point prevalence of neck pain in different countries ranges from 2443.9 to 6151.2 cases per 100,000 population, with the highest values in western Europe [1, 5]. The mean percentages for one-year prevalences and lifetime prevalences of adults worldwide are 37.2% and 48.5%, respectively [6]. Although acute neck pain usually resolves within two months, approximately 50% of patients are not completely pain free one year after an episode of neck pain [7–9]. This illustrates the often chronic-episodic course of the condition, with patients experiencing persistent or recurrent episodes of neck pain [10].

Management of patients with neck pain is a major challenge in physiotherapy, mainly because these patients form a very heterogeneous group in terms of the nature of symptoms, symptom distribution, and underlying pain mechanisms [11]. As neck pain is a multidimensional condition, management should consider multiple factors (e.g. pain mechanisms, and psychological, biological, movement and work-related factors). Among the work-related factors, workload, work or study time, sustained postures or body positions during work and computer work are considered as risk factors for the development of neck pain [1, 12]. The different factors can interact, and their expression may be more or less dominant in each patient, thus influencing the clinical approach [1, 12].

Deficits of sensorimotor capacities (SC) may be one of the factors contributing to neck pain, in particular the persistence or recurrence of neck pain [13]. The sensorimotor system is defined as an integrated whole, comprising afferent and efferent information, with central integration and processing components necessary to provide functional joint stability [14]. It is thought to influence, among others, joint position sense, activation of cervical flexor muscles and control of head-eye movement. The SC of the cervical spine are related to neck pain [15] and patients with neck pain often demonstrate reduced SC, e.g. reduced joint position sense [16–18], altered activation patterns of the cervical muscles [19–21], or disturbed head-eye movement control [22]. Furthermore, the persistence of deficits in the sensorimotor system can continue even after pain relief. It is hypothesized that persistence of these deficits may contribute to some patients experiencing recurrent episodes of neck pain [23–25] and the integration of sensorimotor training in the management of patients with neck pain has shown promising results [13]. Therefore, evaluation of SC in patients with neck pain is important [26]. Various tests to evaluate the sensorimotor system have been developed and are widely used in physiotherapy practice and research. However, the terminology used is often confusing, and there is no consensus on how SC of the neck should be assessed [14, 27]. Systematic reviews of tests for SC of the neck have investigated only a limited selection of tests assessing single aspects of SC, such as joint position sense [28] or muscle function [29–31]. A systematic review, providing a comprehensive overview of all available tests to assess all different aspects of SC of the neck, is lacking.

Given that many tests exist for assessment of SC of the neck, the challenge is to choose the most appropriate test for use in a specific situation. From a scientific perspective, knowledge about the quality of a test, i.e. measurement properties, is important when making this decision. The quality of a test depends on three criteria: reliability, validity and responsiveness [32].

This systematic review investigates the domain reliability. Reliability is the degree to which measurements are free from measurement error. The domain reliability includes three measurement properties: reliability, (expressing the proportion of the total variance in the measurements which is due to ‘true’ differences between patients), measurement error (which is the systematic and random error of a patient’s score that is not attributed to true changes in the construct to be measured), and internal consistency [32]. Internal consistency is usually investigated in self-reporting multi-item questionnaires and therefore is not relevant for the single-item tests used to assess SC.

The aim of this systematic review is to include all tests assessing any aspect of SC of the neck. Therefore, since many different tests are described in the literature, this review focusses only on reliability. Of course, when deciding which test to use, it would also be important to consider the different aspects of validity.

The concepts of reliability and measurement error are related, but focus on different purposes. Reliability focusses on the variability between patients or measurements and is influenced by the variation in the population where the test is used. On the other hand, measurement error is a relevant parameter for measurement of change over time, and it is not affected by population variability [33]. In clinical practice physiotherapists are interested in both concepts. The distinction between patients with and without deficits in the sensorimotor system (diagnostic purpose) is important. But measurement error is also an issue, as change over time, i.e. the evolution of the patient’s symptoms, is of interest.

The aims of this systematic review are: (a) to provide an overview of tests used in physiotherapy to assess SC in patients with neck pain; and (b) to provide information about the reliability and measurement error of these tests, to enable physiotherapists to select appropriate tests.

## Methods

### Design

A meta-analysis of studies investigating the reliability and measurement error of tests assessing SC of patients with neck pain in a physiotherapy setting.

### Search strategy

The databases CINAHL, Embase and PsycINFO were searched up to July 2020 and for Medline up to May 2021. Blocks of search terms were developed for: (a) construct of interest (sensorimotor capacities), (b) population (patients with neck pain), (c) the sensitive PubMed filter developed by Terwee et al. [34] for the identification of studies about measurement properties of measurement instruments, and (d) the exclusion filter proposed by Terwee et al. [34] to exclude irrelevant studies. The two filters were adapted to the other databases, adopting the strategy used by Ammann-Reiffer et al. [35]. There was no language restriction. The reference lists of systematic reviews retrieved were hand searched for further eligible studies. The detailed search strategy is shown in Additional file 1.

### Selection process

**Fig. 1:**
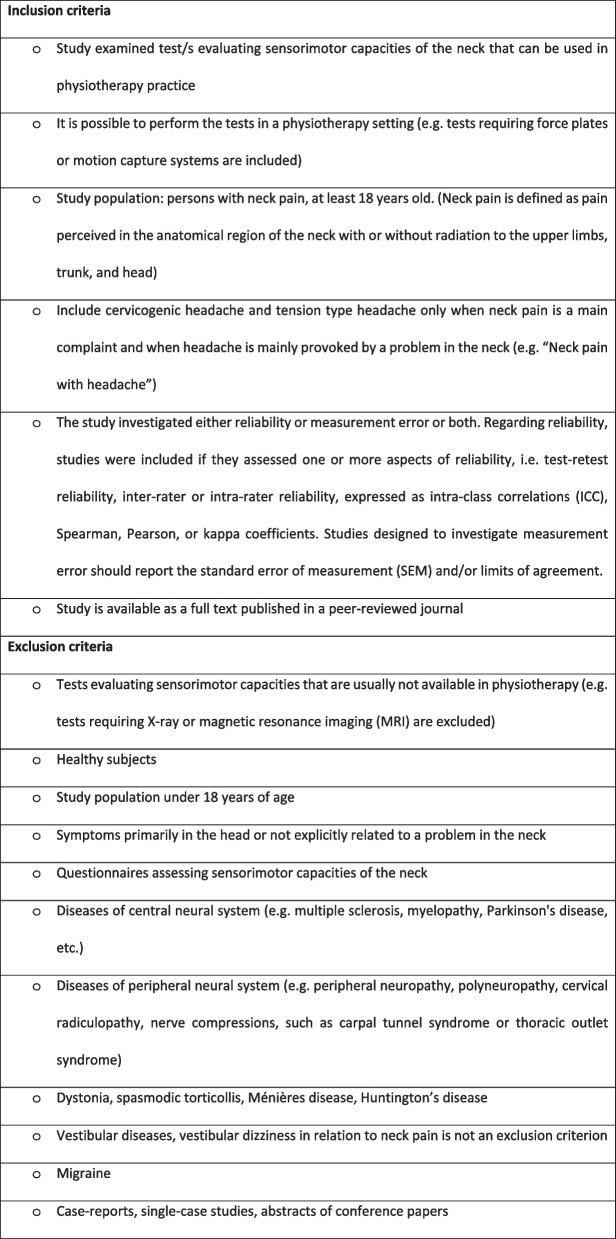
Criteria for inclusion or exclusion of studies

Two reviewers (SE and either RH or MT) screened the titles and abstracts independently, based on the predefined inclusion and exclusion criteria listed in Fig. 1. Disagreements were discussed and, if necessary, a third reviewer (CB) made a decision regarding inclusion. Reviewers in the team were able to read English, German, Dutch, French, Danish and Norwegian, and no exclusion of relevant papers based on language was noted.

Full-text screening was performed independently by two researchers (SE and RH) using the same predefined criteria (Fig. 1). After each screening step (title/abstract and full text), in the case of any disagreement about inclusion, consensus was reached through discussion with a third reviewer (CB). The screening was carried out using Covidence systematic review software [36].

### Data extraction

Data extraction was conducted using REDCap electronic data capture tools hosted at the University of Applied Sciences and Arts Western Switzerland (HES-SO) Valais [37] by SE and RH. The first five studies were checked by a third researcher (CB) to ensure the correct procedure. Data were extracted on study characteristics, reliability, and measurement error of the different tests. Two researchers (SE and RH) assessed methodological quality, applying the COSMIN risk of bias tool in the adapted version for clinician-reported or performance-based outcome measures [38]. Each criterion was rated on a four-point rating system (i.e. very good, adequate, doubtful, or inadequate). The lowest rating determined the overall rating of the study (worst-score-counts method). The detailed tables for risk of bias assessment are shown in Additional file 2 (reliability) and Additional file 3 (measurement error). A third researcher (CB) performed a check of the first studies. Data extraction and synthesis was conducted with all included studies regardless of their methodological quality.

### Data synthesis and analysis

The intraclass correlation coefficients of studies that used the same device and similar instructions for the corresponding test were quantitatively pooled. When pooling was not feasible, the study results were qualitatively summarized, by reporting the lowest and highest values. Because of the large number of tests applied for different directions of movement of the neck (left rotation, right rotation, etc.) test directions were summarized with reliability or measurement error values that led to the same conclusion regarding the criteria for good measurement properties, with the lowest and highest value. Tests directions with very different values (i.e. when the conclusion about the appropriateness of the reliability or the measurement error for this direction would be different from that for other directions) were reported separately.

The overall results for the reliability and/or measurement error of single studies or of summarized or pooled studies were compared against the criteria for good measurement properties. In a next step, the quality of evidence was graded according to the modified GRADE method proposed by the COSMIN group [38]. The quality of evidence was classified as high, moderate, low, or very low. The score was downgraded for risk of bias (minus one for serious, minus two for very serious and minus three for extremely serious risk of bias), inconsistency (minus one if more than one study per test available I^2^ > 0.5), and imprecision (minus one if total sample size n = 50–100, minus two if total simple size n < 50). The score was not downgraded for indirectness, due to the restrictive inclusion criteria used in the current study [38]. Detailed tables of the quality of evidence criteria are shown in Additional file 4 (reliability) and Additional file 5 (measurement error).

## Results

**Fig. 2:**
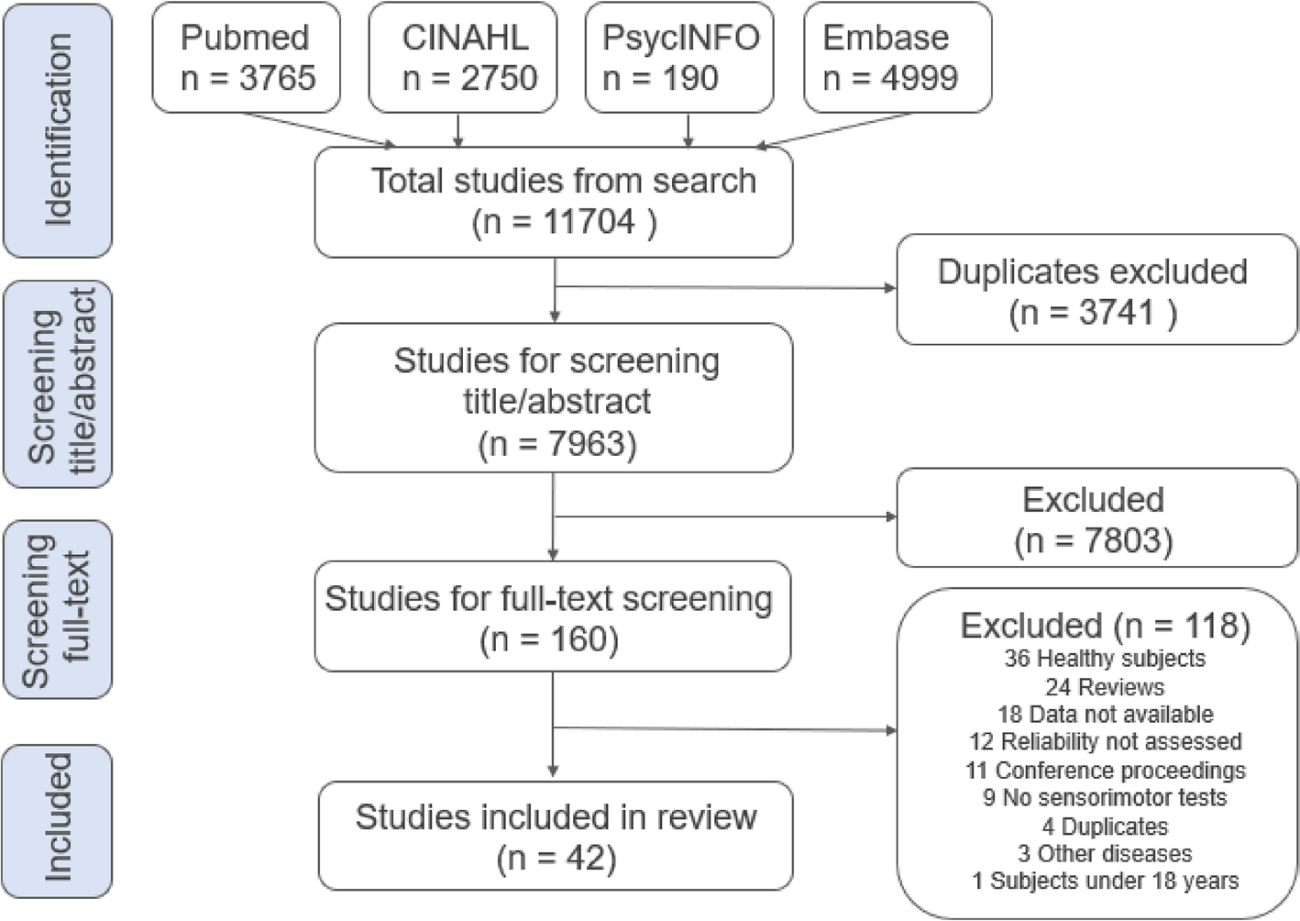
Flow chart

In total 11,704 studies were found using the search strategy in four databases (Medline, CINAHL, Embase, PsycINFO). First, 3741 duplicates were removed. The remaining 7963 studies were screened for title and abstract, and 7803 were excluded based on the predefined criteria. Of the 160 full-text studies, 118 were excluded. The reasons for exclusion are listed in Fig. 2.

A final total of 42 studies, investigating a total of 206 tests for the assessment of SC in patients with neck pain, were included in the systematic review (Table 1).

**Table 1:** Characteristics of the included studies

Study	Population	Age, mean (sd)	Women n (%)
Chiu (2002) [39]; China	Subjects with mechanical neck pain	27.0 (9.5)	12 (57%)
Cibulka (2017) [40]; USA	Healthy adults with mild neck pain gathered through flyers, email, and word of mouth	22.8 (3.5)	23 (62%)
Cleland (2006) [41]; USA	Patients with mechanical neck pain referred to physical therapy at the Rehabilitation Services of a hospital	41 (12.9)	18 (82%)
De Pauw (2020) [42]; Belgium	Patients reporting neck pain, recruitment by advertising on social media and distribution of flyers	28.0 (8.2)	17 (68%)
Dvir (2006) [43]; Israel	Patients injured in whiplash-type accidents	37.1 (9.9)	13 (52%)
Edmondston (2008) [44]; Australia	Subjects with postural neck pain, recruited through poster advertising and through a university physical therapy clinic	36 (11)	14 (67%)
Fletcher (2008) [45]; USA	Subjects with neck pain from a college campus and a community setting	33.6 (10.3)	15 (68%)
Ghorbani (2020) [46]; Iran	Participants with neck pain from the University of Medical Sciences, invited via word of mouth	25.9 (1.04)	13 (65%)
Gonçalves (2019) [47]; Portugal	Individuals with neck pain recruited from a private clinical practice and from the general population	43.6 (13.3)	26 (79%)
Grod (2002) [48]; Canada	Patients with chronic neck pain from two chiropractic offices	38.5 (NA)	11 (58%)
Hanney (2014) [49]; USA	Patients with mechanical neck pain who presented to clinics	48.9 (14.8)	14 (64%)
Harris (2005) [50]; USA	Subjects with neck pain	38 (10)	61%^a^
Hoppenbrouwers (2006) [51]; Netherlands	Patients with neck pain from three physical therapy practices	43.0 (10.9)	15 (60%)
Hoving (2005) [52]; Netherlands	Patients with neck pain, referred by general practitioners for physical therapy	45.5 (9.2)	20 (63%)
Kristjansson (2004) [53]; Iceland	Female patients with chronic whiplash (grades I or II of Quebec Task Force classification) recruited from physiotherapy clinics	30.0 (8.8)^a^	10 (100%)
Kristjansson (2010) [54]; Iceland	Subjects with non-traumatic neck pain	38.0 (8.3)	11 (61%)
Kumbhare (2005) [55]; Canada	Patients with WDA (grade II of Quebec Task Force classification) recruited from a hospital	39.9 (14.9)	49 (69%)
Law (2013) [56]; China	Patients with neck pain from the out-patient Physiotherapy Department of a hospital	44.52 (7.11)	17 (65%)
Lourenço (2016) [57]; Portugal	Students with idiopathic neck pain from a university	20.18 (1.84)	17 (77%)
Majcen Rosker (2021) [58]; Slovenia	Patients with chronic neck pain, referred by an orthopaedic surgeon	46.2 (4.8)	23 (72%)
Martins (2018) [59]; Portugal	Participants with neck pain recruited from the general population	36.8 (2.4)	28 (85%)
Murphy (2010) [60]; New Zealand	Subjects with chronic neck pain recruited through advertisements in local papers and word of mouth	44.8 (8.5)	11 (79%)
O'Leary (2005) [61]; Australia	Subjects with neck pain recruited by printed and electronic advertising within the University	27.9^a^	75%^a^
Pearson (2009) [62]; Canada	Patients with WAD recruited from a rehabilitation and return-to-work program and from advertisements in local newspapers	36.6 (10.8)	6 (43%)
Peolsson (2007) [63]; Sweden	Patients with chronic neck disorders from primary care and from private clinicians	intra-rater: 49 (11); inter-rater: 47 (8)	intra-rater: 9 (90) ; inter-rater: 6 (75)
Petersen (2000) [64]; USA	Subjects with present complaints of local cervical pain	40.2 (8.7)	13 (65%)
Piva (2006) [65]; USA	Patients referred to a University Spine Speciality Centre with a primary complaint of neck pain	41 (12)	18 (60%)
Pourahmadi (2018) [66]; Iran	Subjects with non-traumatic neck pain recruited by purposive and snowball sampling	31.12 (6.38)	20 (50%)
Rheault (1992) [67]; USA	Subjects with a history of cervical spine pathology	37.41 (14.1)	15 (68%)
Röijezon (2010) [68]; Schweden	Women with non-traumatic neck pain recruited by advertising in local papers and by information to job holders	48 (7)	16 (100%)
Roren (2009) [69]; France	Patients with neck pain from a rehabilitation department	54.7 (14.2)	23 (56%)
Schneider (2013) [70]; Canada	Patients with persistent neck pain, referred to a tertiary interventional pain management centre	46 (NA)	37 (66%)
Sebastian (2015) [71]; USA	Patients with a diagnosis of neck pain	Range 30-75y	NA
Shahidi (2012) [72]; USA	Participants with neck pain recruited from a university medical campus and surrounding community	34.9 (9.9)	9 (47%)
Stenneberg (2018) [73]; Netherlands	Patients with neck pain recruited from five primary care physical therapy practices	45.2 (15.3)	19 (73%)
Sterling (2002) [74]; Australia	Patients with chronic neck pain of traumatic or non-traumatic origin, recruited via written advertisement within a university	31.63 (11.5)	13 (68%)
Uddin (2013) [75]; Canada	Patients with mechanical neck disorder	45.43 (11.88)^b^	81%^b^
Vernon (1992) [76]; Canada	Subjects presenting to the problem case clinic of a chiropractic college teaching clinic (mechanical neck pain syndrome, whiplash-type cervical strain injury)	37.5 (8.6)	12 (50%)
Werner (2018) [77]; Switzerland	Subjects with neck pain (non-specific or WAD) attending the physiotherapy department of a hospital	40.1 (12.35)	13 (65%)
Williams (2012) [78]; United Kingdom	Patients following a whiplash injury, attending an Emergency Department	41 (14.8)	13 (68%)
Williams (2012) [78]; United Kingdom	Patients following a whiplash injury, attending an Emergency Department	38 (11.3)	19 (50%)
Ylinen (2004) [79]; Finland	Women with nonspecific chronic neck pain, recruited through local occupational health care services	44 (6)	21 (100%)
Youdas (1991) [80]; USA	Patients referred to a Clinic Department of Physical Medicine and Rehabilitation with orthopaedic disorders	59.1 (15.7)	39 (65%)

*WAD* Whiplash associated disorders, *NA* Not available

^a^values only for total number of participants (participants with and without neck pain)

^b^values for all 106 patients, reliability was only calculated for 34 patients with unknown age and gender distribution

**Fig. 3:**
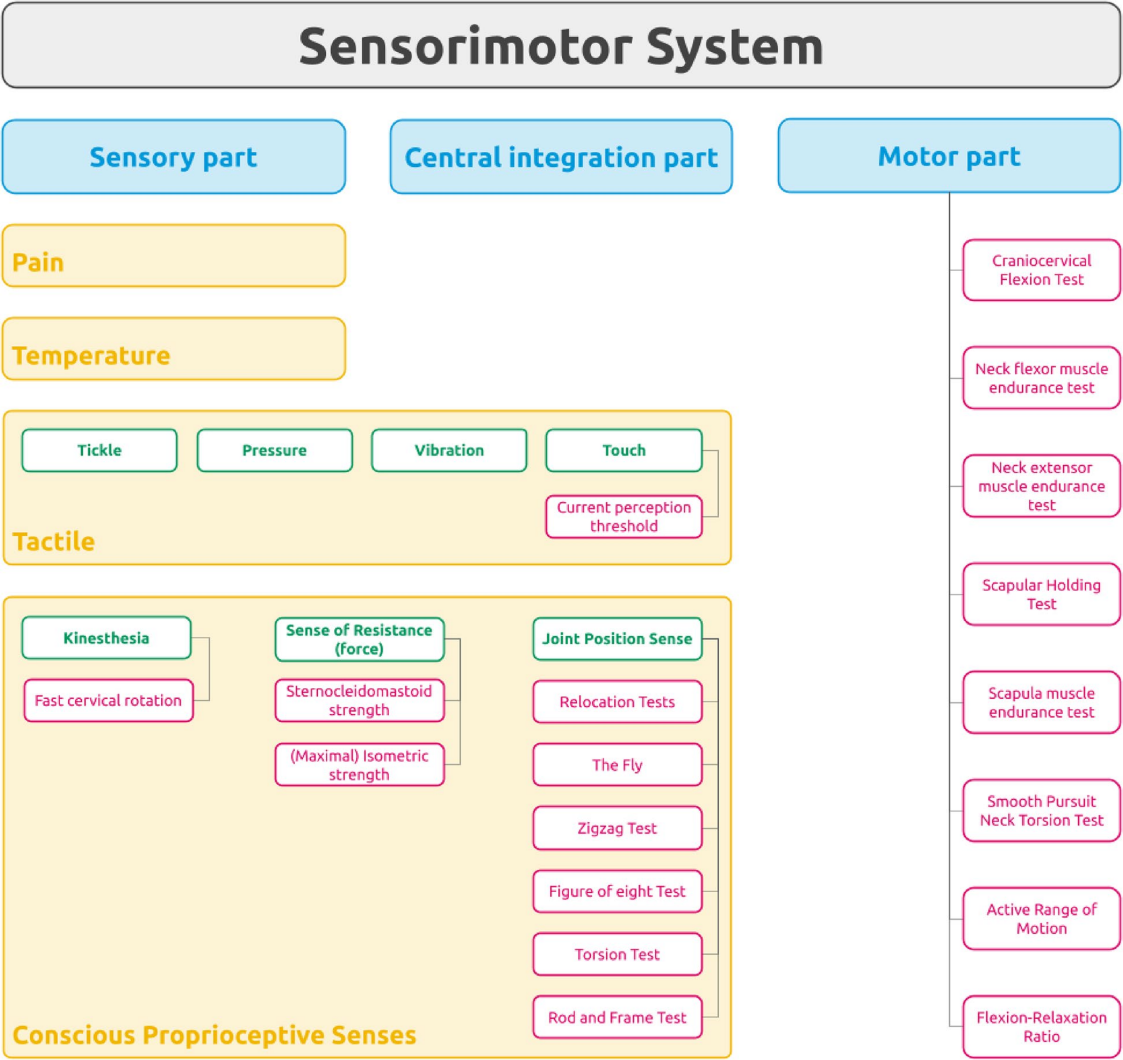
Sensorimotor system definition (according to Riemann & Lephart 2002 (14)) and the 18 groups of tests included in this systematic review* (pink boxes)*

Tests were categorized into 18 different groups (e.g. tests for active range of motion in the different movement directions of flexion, extension, lateral flexion, and rotation with the help of different devices were grouped together as active range of motion tests). Based on the classification of Riemann & Lephart [14], tests for the sensory and the motor components of the sensorimotor system were identified, but no tests for the central integration component were found. Within the sensory component, tests in the subcomponents “tactile” and “conscious proprioceptive senses” were found. As this study did not search for tests assessing pain, the subcomponent “pain” does not contain a test. A list of all groups of tests is shown in Fig. 3.

According to the COSMIN criteria the following 12 tests were rated as good: craniocervical flexion test (test-retest reliability), neck flexor muscle endurance test (inter-rater and test-retest reliability), neck extensor muscle endurance test (inter-rater and test-retest reliability), sternocleidomastoid muscle strength (test-retest reliability), maximal voluntary isometric contraction (test-retest reliability), isometric strength with the help of different devices (test-retest reliability), flexion-relaxation ratio (test-retest reliability), active range of motion test with the help of different devices (inter-rater and test-retest reliability), figure of eight test (inter-rater and test-retest reliability), zigzag test (inter-rater and test-retest reliability), smooth pursuit neck torsion test (test-retest reliability), and rod and frame test (test-retest reliability). An overview of the ratings of all tests is shown Table 2. However, regarding reliability, the quality of evidence was rated as low or very low for all included studies. The reasons for downgrading are shown in Additional file 4.

**Table 2:** Summary of findings

Test	Pooled^1^	Test-retest	Reliability Index (95% CI), (rating of criteria for good reliability)	Quality of the evidence (modified GRADE) Reliability	Quality of the evidence (modified GRADE)	SDC (rating of criteria for good ME)	Quality of the evidence (modified GRADE) ME	Quality of the evidence (modified GRADE) ME
Craniocervical Flexion Test adapted (De Pauw ,2020) [42]		inter-rater	0.64 (0.33-0.82), (-)	+	Very Low	2.72 (?)	++	Low
Craniocervical Flexion Test adapted (De Pauw, 2020) [42]		test-retest	0.65 (0.35-0.83), (-)	+	Very Low	2.58 (?)	++	Low
Craniocervical Flexion Test muscle activation		test-retest	0.7 (0.39-0.86), (+)	+	Very Low	4.96 (?)	++	Low
Craniocervical Flexion Test muscle endurance		test-retest	0.9 (0.8-0.95), (+)	+	Very Low	2.49 (?)	++	Low
Neck flexor muscle endurance Test	x	inter-rater	0.75 (0.29-0.93), (+)	++	Low	6.38 to 31.88 (?)	++	Low
Neck flexor muscle endurance Test	x	test-retest	0.84 (0.68-0.92), (+)	+	Very Low	17.74 to 23.01 (?)	+	Very Low
Neck extensor muscle endurance Test	x	inter-rater	0.84 (0.69-0.92), (+) sic!	++	Low			
Neck extensor muscle endurance Test	x	test-retest	0.84 (0.69-0.92), (+)	++	Low	2.05 to 71.51 (?)	+	Very Low
Scapula muscle endurance Test Standing		test-retest	0.67 (0.31-0.85), (-)	+	Very Low	30.21 (?)	++	Low
Scapular Holding Test adapted (summarized left and right)		inter-rater	0.54-0.63 (0.19-0.82), (-)	+	Very Low	3.02-3.3 (?)	++	Low
Scapular Holding Test adapted (summarized left and right)		test-retest	0.68-0.7 (0.41-0.86), (- to +)	+	Very Low	2.44-3.08 (?)	++	Low
Sternocleidomastoid strength (summarized left and right)		test-retest	0.95-0.97 (0.91-0.99), (+)	+	Very Low	4.63-5.04 (?)	++	Low
Isometric Muscle Strength Lower Trapezius (summarized left and right)		inter-rater	0.65-0.78 (0.28-0.91), (- to +)	+	Very Low			
Isometric Muscle Strength Middle Trapezius, Rhomboid (summarized left and right)		inter-rater	0.33-0.59 (-0.07-0.82), (-)	+	Very Low			
Maximal voluntary isometric contraction (six parameters summarized)		test-retest	0.76-0.91 (NA), (+)	+	Very Low	0.03-26.06 (?)	++	Low
Max isometric strength Flex outer torque Dorsal Head Force		test-retest	0.09 (NA-NA), (-)	+	Very Low	141.36 (?)	++	Low
Isometric strength Dynamometer		inter-rater	0.39-0.72 (-0.1-0.89), (- to +)	+	Very Low			
Isometric strength (Dynamometer, Modified Sphygmomanometer, Multi Cervical Rehabilitation Unit, Neck strength measurement system)	x	test-retest	0.74-0.99 (0.47-1), (+)	+	Very Low	1.94-54.33 (?)	+ to ++	Very Low to Low
Flexion-relaxation ratio		test-retest	0.83 (0.67-0.92), (+)	+	Very Low			
AROM visual estimation (six directions summarized)		inter-rater	0.42-0.82 (NA), (- to +)	+	Very Low			
AROM Universal Goniometer (all directions)	x	inter-rater	0.66-0.82 (0.47-0.93), (- to +)	+ to ++	Very Low to Low	3.41-15.25 (?)	+ to ++	Very Low to Low
AROM Universal Goniometer (all directions)	x	test-retest	0.71-0.89 (0.5-0.97), (+)	+ to ++	Very Low to Low	2.22-12.2 (?)	+ to ++	Very Low to Low
AROM Universal Goniometer Rot (left and right summarized)		test-retest	0.31 (-0.12-0.64)^2^, (-)	+	Very Low	27.5 (?)	++	Low
AROM Electronic Goniometer (three directions summarized)		inter-rater	0.81-0.86 (0.62-0.94), (+)	+	Very Low	14.91-22.23 (?)	++	Low
AROM Electronic Goniometer (three directions summarized)		test-retest	0.89-0.92 (0.77-0.96), (+)	+	Very Low	11.28-16.58 (?)	++	Low
AROM Gravity Goniometer (eight movements summarized)		inter-rater	0.74-0.89 (0.26-0.95), (+)	+	Very Low	8.87-15.52 (?)	+ to +++	Very Low to Moderate
AROM Inclinometer (three directions summarized)		test-retest	0.41-0.75 (-0.16-0.89), (- to +)	+	Very Low	14.8-22.06 (?)	++	Low
AROM Digital inclinometer (six directions summarized)		inter-rater	0.73-0.89 (0.05-0.96), (+)	+	Very Low			
AROM Digital inclinometer (six directions summarized)		test-retest	0.53-0.84 (-0.32-0.94), (- to +)	+	Very Low	7.45-17.52 (?)	++	Low
AROM Digital inclinometer EDI-320 (three directions summarized)		inter-rater	0.89-0.95 (0.77-0.98), (+)	+	Very Low			
AROM Digital inclinometer EDI-320 (three directions summarized)		test-retest	0.93-0.96 (0.86-0.98), (+)	+	Very Low			
AROM Gravity Inclinometer (four pooled directions summarized)	x	inter-rater	0.74-0.86 (0.38-0.95), (+)	+	Very Low	8.59-19.4 (?)	+	Very Low
AROM Gravity Inclinometer (four directions summarized)		test-retest	0.91-0.95 (0.85-0.97), (+)	+	Very Low	6.65-8.04 (?)	++	Low
AROM CROM device (six pooled directions summarized)	x	inter-rater	0.82 - 0.93 (0.73-0.97), (+)	+ to ++	Very Low to Low	10.26-18.02 (?)	+	Very Low
AROM CROM device (six pooled directions summarized)	x	test-retest	0.91-0.95 (0.68-0.98), (+)	+ to ++	Very Low to Low	3.6-11.36 (?)	++	Low
AROM Fastrak (six directions summarized)		test-retest	0.64-0.88 (NA), (- to +)	+	Very Low	6.54-18.63 (?)	++	Low
AROM Multi Cervical Rehabilitation Unit (six directions summarized)		test-retest	0.82-0.96 (0.66-0.98), (+)	+	Very Low			
AROM OSI Spine Motion Analyser (six directions summarized)		test-retest	0.68-0.96 (NA), (- to +)	+	Very Low	5.13-9.12 (?)	+	Very Low
AROM Zebris (six directions summarized)		test-retest	0.81-0.86 (NA), (+)	+	Very Low	15.52-28.27 (?)	++	Low
AROM iPhone (six pooled directions summarized)	x	inter-rater	0.74-0.96 (0.39-0.98), (+)	+ to ++	Very Low to Low	4.1-9.67 (?)	+	Very Low
AROM iPhone (six pooled directions summarized)	x	test-retest	0.69-0.84 (0.43-0.96), (- to +)	+ to ++	Very Low to Low	2.86-12.5 (?)	+ to ++	Very Low to Low
AROM Android (four directions summarized)		inter-rater	0.9-0.92 (0.72-0.97), (+)	+	Very Low			
AROM Android (four directions summarized)		test-retest	0.85-0.9 (0.61-0.96), (+)	+	Very Low	6.9-12.16 (?)	++	Low
AROM Android Rotation (left and right summarized)		inter-rater	0.17-0.48 (-0.98-0.8), (-)	+	Very Low			
AROM Android Rotation (left and right summarized)		test-retest	0.13-0.52 (-1.42-0.82), (-)	+	Very Low	24.05-32.89 (?)	++	Low
Relocation Test (Rotation pooled, seven movements summarized)	x	test-retest	0.62-0.85 (0.32-0.93), (- to +)	+ to ++	Very Low to Low	3.33-4.16 (?)	+ to ++	Very Low to Low
The Fly (twelve parameters summarized)		test-retest	0.58-0.86 (0.38-0.86), (- to +)	+	Very Low			
The Fly Overshoots (three level summarized)		test-retest	0.14-0.42 (-0.14-0.62), (-)	+	Very Low			
Torsion Test (left and right summarized)		test-retest	0.58.0.71 (0.14-0.85), (- to +)	+	Very Low	2.77-4.43 (?)	++	Low
Figure of eight Test (six parameters summarized)		inter-rater	0.76-1 (0.62-1), (+)	+	Very Low	0.06-5.6 (?)	++++	High
Figure of eight Test (four parameters summarized)		test-retest	0.81-1 (0.64-1), (+)	+	Very Low	0.03-8.09 (?)	++++	High
Zigzag Test (six parameters summarized)		inter-rater	0.8-1 (0.6-1), (+)	+	Very Low	0.08-2.27 (?)	++++	High
Zigzag Test (four parameters summarized)		test-retest	0.95-1 (0.78-1), (+)	+	Very Low	0.03-1.64 (?)	++++	High
Smooth Pursuit Neck Torsion Test (diff) Amplitude 40°, Velocity 20°/s		test-retest	0.75 (0.44-0.81), (+)	+	Very Low	0.11 (?)	++	Low
Rod and Frame Test (three parameters summarized)		test-retest	0.74-0.9 (NA), (+)	+ to ++	Very Low to Low			
Fast cervical rotation (five directions summarized)		test-retest	0.37-0.86 (-0.16-0.95), (- to +)	+	Very Low	0.3-91.47 (?)	++	Low
Fast cervical rotation Conjunct movements		test-retest	-0.07 (-0.54-0.45), (-)	+	Very Low	18.02 (?)	++	Low
Current perception threshold (nine parameters summarized)		test-retest	0.47-0.86 (0.08-0.93), (- to +)	+	Very Low			

*Summarized* Smallest and highest ICC from several tests, *CI* Confidence interval, *SDC* Smallest detectable change, *ME* Measurement error, *AROM* Active range of motion

Criteria for good reliability: + (sufficient) if ICC or (weighted) Kappa at least 0.7; ? (indeterminate) if ICC or (weighted) Kappa not reported; - (insufficient) if ICC or (weighted) Kappa < 0.7.

Criteria for good measurement error: + (sufficient) if smallest detectable change (SDC) or Limits of Agreement (LoA) or Coefficient of Variation (CV)*√2*1.96 < Minimal Clinically Important Change (MCIC); ? (indeterminate) if MCIC was not defined; - (insufficient) if SDC or LoA or CV*√2*1.96 >= MCIC. (37)

Quality of evidence: High: we are very confident that the true measurement property lies close to that of the estimate of the measurement property; Moderate: we are moderately confident in the measurement property estimate: the true measurement property is likely to be close to the estimate of the measurement property, but there is a possibility that it is substantially different; Low: our confidence in the measurement property estimate is limited: the true measurement property may be substantially different from the estimate of the measurement property; Very low: we have very little confidence in the measurement property estimate: the true measurement property is likely to be substantially different from the estimate of the measurement property (38).

Regarding measurement error, the criteria for good measurement error were rated as unknown for all included tests, because the minimal clinically important change was not reported. The quality of evidence was rated very low to high (Table 2). Reasons for downgrading are shown in Additional file 5.

## Discussion

This systematic review included 42 studies evaluating 206 tests, with the aim of investigating the reliability and measurement error of tests for SC in patients with neck pain. The main findings are, firstly, that tests for the sensory and motor components of the sensorimotor system were found, but not for the central integration component. Furthermore, no data were found on reliability or measurement error in patients with neck pain for some tests that are used in practice, such as the movement control tests, which would belong to the motor component; secondly, approximately half of the tests, particularly tests that are easier to standardize with regard to test position or movement direction, showed good reliability; and, finally, tests evaluating more complex movements, which are more difficult to standardize, were less reliable.

In general, all included muscle endurance tests, had good (relative) reliability values according to the criteria for good measurement properties proposed by COSMIN, except for the scapula muscle endurance test in standing position. The execution of this test is much more complex and more difficult to standardize than other tests. Furthermore, scapula movements, compensatory movements, muscle recruitment etc. are more difficult to assess compared with neck movements where the movement directions follow the sagittal, frontal, or transversal plane in a more stable way. Similarly, regarding reliability of the isometric muscle strength tests, tests involving the judgement of movements or muscle recruitment around the scapula have lower values for reliability than tests for isometric activity of the head into flexion, extension, lateral flexion, or rotation. Again, this may be because scapula positions are more difficult to standardize, and isometric contractions of the scapula muscles are more difficult to assess regarding compensatory movements than isometric muscle activity of the muscles of the cervical spine.

The test of fast cervical rotations showed very low reliability, possibly due to the very complex characteristics of these movements, which make it difficult to standardize the test. The tests assessing active range of motion (AROM) of the cervical spine showed that assessment of rotation is more difficult compared with the other movement directions. This is particularly evident when the rotation is assessed as a single movement (combined right and left values) and when AROM is assessed with the help of a smartphone. In the current analysis, the values were less reliable for Android phones than for iPhones (see Table 2). This could be due to differences in the study protocols. In the study that used an Android phone, it was only held against the head, whereas in the study assessing AROM with an iPhone, the device was fastened securely to the forehead with a rigid strap, which might produce more reliable results. The assessment of AROM with the help of a dynamometer or goniometer showed good test-retest reliability results, but less good values for inter-rater reliability. It is evident that good values for inter-rater reliability are more difficult to achieve, because more sources of variation are included (e.g. different testers). Thus, the standardization of these types of tests is often a problem.

Using the example of the craniocervical flexion test (CCFT), this review shows that tests that require a substantial subjective rating (e.g. judgement of muscle recruitment or movement patterns) lead to lower reliability compared with more objective criteria (e.g. time). The current results are in line with a recent systematic review by Selistre and colleagues [30], investigating clinical tests for measuring strength or endurance of cervical muscles. They found moderate to good intra- and inter-rater reliability for the CCFT, cervical flexor endurance test, cervical extensor endurance test and cervical muscle strength assessed using a handheld dynamometer. The results of the current review are comparable for the CCFT, the cervical flexor endurance test and the cervical extensor endurance test. For the cervical muscle strength tests, the current review performed a more detailed analysis, e.g. Selistre et al. [30] described the cervical strength tests only with the handheld dynamometer and not with other devices. In the current study, the cervical strength tests with dynamometer showed good results for test-retest reliability, but poorer results for intra-rater reliability. The results of the current review are also comparable with those of a recent systematic review of the measurement properties of the CCFT [31]. The authors classified the inter-rater and the intra-rater reliability of the CCFT as positive and the level of evidence as moderate. The measurement error was classified as indeterminate and the level of evidence as unknown. The authors identified the same problems as found in the current review, such as low methodological quality of the included studies and missing data on minimal clinically important change.

The two recent systematic reviews on measurement properties of tests for the SC of the neck included studies with participants with and without neck pain [30, 31]. Both stated that studies on participants with neck pain were lacking, which is in line with the current results. The current review excluded several studies because the results for participants with neck pain were not reported separately but only together with those for people without neck pain. It was decided to include only studies with data for patients with neck pain, given our interest in the use of the tests in a clinical setting. Because the reliability of a test is influenced by the heterogeneity of the population in which the test is performed, it is important to know the reliability for a comparable population to that in which the test will be administered. It was also surprising that tests such as the CCFT, which is widely used in clinical practice, are so rarely investigated in patients with neck pain.

The major strength of this study is that it included all available tests for assessment of all aspects of sensorimotor control of the neck. However, the study also has a number of limitations. Many tests were performed only on healthy participants or in a mixed group of participants with and without neck pain. Several studies were excluded, including all studies assessing tests for movement control of the neck, as the authors did not report separate data for the patient group. Secondly, the quality of evidence was low to very low regarding reliability for all included studies. It was necessary to downgrade the level of evidence, mainly because of high risk of bias, inconsistency, and low precision. In the assessment of risk of bias, the item “patient stability” is one of the items that was particularly rated as doubtful in many cases. COSMIN recommends that patient stability should only be rated as very good if the study explicitly describes that the patients’ condition did not change between measurements. As this information was often missing, the current review had to rate the patient stability item as doubtful, even though the time interval between measurements was adequate. A further limitation of this review is that the included studies did not report data on interpretability and feasibility of the different tests, which would be important information for the recommendation of specific tests. Finally, this review did not assess aspects of validity, which would certainly also be important for the selection of appropriate tests.

Better studies are needed on reliability, measurement error and validity of tests in patients with neck pain, because the quality of evidence of the existing research is mainly low or very low, and the reliability of some tests (e.g. for movement control) was not evaluated in patients with neck pain at all.

## Conclusion

Despite the large number of tests available, the quality of evidence is not yet high enough to conclusively inform clinicians which test to use to assess SC in patients with neck pain.

For clinical practice, this systematic review shows that tests with objective criteria and a thorough standardization should be chosen to ensure higher reliability.

Measurement error could not be evaluated because the minimal clinically important change was not available for all tests.

### Supplementary Information

**Additional file 1.** Search strategy (for Medline). Search strategy for Medline

**Additional file 2.** Risk of bias Reliability. Items of the assessment of risk of bias for reliability

**Additional file 3.** Risk of bias Measurement error. Items of the assessment of risk of bias for measurement error

**Additional file 4.** GRADE Downgrading Reliability. Reasons for downgrading

**Additional file 5.** GRADE Downgrading Measurement error. Reasons for downgrading

## Data Availability

All data generated or analysed during this study are included in this published article and its supplementary information files.

## Abbreviations

*AROM*: Active range of motion
*CCFT*: Craniocervical flexion test
*CINAHL*: Cumulative Index to Nursing and Allied Health Literature
*COSMIN*: Consensus-based Standards for the selection of health Measurement INstruments
*Embase*: Excerpta Medica dataBASE
*GRADE*: Grading of Recommendations Assessment, Development and Evaluation
*ICC*: Intra-class correlations
*Medline*: Medical Literature Analysis and Retrieval System Online
