# Supplementary material for: Reliability and measurement error of sensorimotor tests in patients with neck pain: a systematic review
Source: Arch Physiother. 2023 Aug 15;13:15. doi: 10.1186/s40945-023-00170-9 (PMC10428553; doi:10.1186/s40945-023-00170-9)
Supplement: Supplementary file 1 — Additional file 1. Search strategy (for Medline). Search strategy for Medline [file 40945_2023_170_MOESM1_ESM.docx]

| **Additional file 1. Search strategy**  **(for Medline)** | |  |  |
| --- | --- | --- | --- |
| **#1** | **construct search** |  |  |
|  | movement control | movement control[tiab] | OR |
|  |  | motor control[tiab] | OR |
|  |  | control impairment[tiab] | OR |
|  |  | movement impairment[tiab] | OR |
|  |  | movement system impairment[tiab] | OR |
|  |  | relative flexibility[tiab] | OR |
|  |  | sensorimotor[tiab] | OR |
|  |  | sensori-motor[tiab] | OR |
|  |  | kinaesthesia[tiab] | OR |
|  |  | kinaesthetic[tiab] | OR |
|  |  | kinesthesia[tiab] | OR |
|  |  | kinesthetic[tiab] | OR |
|  |  | alignment[tiab] | OR |
|  |  | malalignment[tiab] | OR |
|  |  | posture[tiab] | OR |
|  |  | postural control[tiab] | OR |
|  | vibration | vibration[tiab] | OR |
|  |  | vibratory[tiab] | OR |
|  |  | vibration sense[tiab] | OR |
|  |  | pallesthesia[tiab] | OR |
|  | PPDT | Threshold to detection of passive motion[tiab] | OR |
|  |  | Pressure pain detection threshold[tiab] | OR |
|  |  | Pressure pain threshold[tiab] | OR |
|  |  | PPDT[tiab] | OR |
|  |  | PPDTs[tiab] | OR |
|  |  | PPT[tiab] | OR |
|  |  | threshold detection[tiab] | OR |
|  | TPD | two-point discrimination[tiab] | OR |
|  |  | two point discrimination[tiab] | OR |
|  |  | perception[tiab] | OR |
|  | muscle | muscle endurance[tiab] | OR |
|  |  | muscle activation[tiab] | OR |
|  |  | craniocervical flexion[tiab] | OR |
|  |  | CCFT[tiab] | OR |
|  | position sense | position sense[tiab] | OR |
|  |  | repositioning error[tiab] | OR |
|  |  | reposition acuity[tiab] | OR |
|  |  | reposition sense[tiab] | OR |
|  |  | motion sense[tiab] | OR |
|  |  | movement sensation[tiab] | OR |
|  |  | relocation[tiab] | OR |
|  |  | relocation error[tiab] | OR |
|  |  | Smooth Pursuit Neck Torsion Test[tiab] | OR |
|  |  | SPNTT[tiab] | OR |
|  |  | subjective visual vertical[tiab] | OR |
|  |  | SVV[tiab] | OR |
|  | head-eye coordination | head-eye coordination[tiab] | OR |
|  |  | head eye coordination[tiab] | OR |
|  |  | head-eye co-ordination[tiab] | OR |
|  |  | head eye co-ordination[tiab] | OR |
|  |  | head-eye movement control[tiab] | OR |
|  |  | head eye movement control[tiab] | OR |
|  |  | eye movement[tiab] | OR |
|  |  | oculomotor control[tiab] | OR |
|  | cortical representation of body schema | recognise[tiab] | OR |
|  |  | recognize[tiab] | OR |
|  |  | laterality judgment[tiab] | OR |
|  |  | graphaesthesia[tiab] | OR |
|  |  | cortical representation[tiab] | OR |
|  | general terms | coordination[tiab] | OR |
|  |  | proprioception[tiab] | OR |
|  |  | neuromuscular control[tiab] | OR |
|  |  | instability[tiab] | OR |
|  |  | joint stability[tiab] | OR |
|  |  | postural stability[tiab] | OR |
|  |  | shifting[tiab] | OR |
|  |  | shift[tiab] | OR |
|  |  | “range of motion”[tiab] | OR |
|  |  | (neck[tiab] AND strength[tiab]) | OR |
|  |  |  |  |
| **#2** | **population search** | #a AND #b = #c |  |
|  |  | #c OR #d |  |
|  | a) | neck[tiab] | OR |
|  |  | cervical[tiab] | OR |
|  |  | upper spine[tiab] | OR |
|  |  | cervicogenic[tiab] | OR |
|  | b) | pain[tiab] | OR |
|  |  | ache[tiab] | OR |
|  |  | disorder[tiab] | OR |
|  |  | disorders[tiab] | OR |
|  |  | symptom[tiab] | OR |
|  |  | symptoms[tiab] | OR |
|  |  | syndrome[tiab] | OR |
|  |  | syndromes[tiab] | OR |
|  |  | problem[tiab] | OR |
|  |  | problems[tiab] | OR |
|  |  | impairment[tiab] | OR |
|  |  | impairments[tiab] | OR |
|  | d) | cervicalgia[tiab] | OR |
|  |  | torticollis[tiab] | OR |
|  |  | cervicogenic headache[tiab] | OR |
|  |  | "wry neck"[tiab] | OR |
|  |  | dysfunction[tiab] | OR |
|  |  |  |  |
| **#3** | **#1 AND #2** |  |  |
|  |  |  |  |
| **#4** | NOT (nach construct und population) | fracture[tiab] | OR |
|  |  | fractures[tiab] | OR |
|  |  | orthodontic[tiab] | OR |
|  |  | bite[tiab] | OR |
|  |  | cervical spine cord injury[tiab] | OR |
|  |  | cervical spinal cord injury[tiab] | OR |
|  |  | Meniere's disease[tiab] | OR |
|  |  | Meniere[tiab] | OR |
|  |  | Bow Hunter's[tiab] | OR |
|  |  | Malignancies[tiab] | OR |
|  |  | cancer[tiab] | OR |
|  |  | oncology[tiab] | OR |
|  |  | oncologic[tiab] | OR |
|  |  | malignancy[tiab] | OR |
|  |  | bladder neck[tiab] | OR |
|  |  | plagiocephaly[tiab] | OR |
|  |  | pediatric[tiab] | OR |
|  |  |  |  |
| #5 | **#3 NOT #4** |  |  |
| **#6** | **#5 AND filter for measurement properties** | |  |
| **#7** | **#6 NOT exclusion filter** | |  |

**Filter for measurement properties** (Terwee et al., 2009)

(((instrumentation OR methods OR Validation Studies OR Comparative Study[pt] OR "psychometrics"[MeSH] OR psychometr*[tiab] OR clinimetr*[tw] OR clinometr*[tw] OR "outcome assessment (health care)"[MeSH] OR outcome assessment[tiab] OR outcome measure*[tw] OR "observer variation"[MeSH] OR observer variation[tiab] OR "Health Status Indicators"[Mesh] OR "reproducibility of results"[MeSH] OR reproducib*[tiab] OR reliab*[tiab] OR unreliab*[tiab] OR valid*[tiab] OR coefficient[tiab] OR homogeneity[tiab] OR homogeneous[tiab] OR "internal consistency"[tiab] OR (cronbach*[tiab] AND (alpha[tiab] OR alphas[tiab])) OR (item[tiab] AND (correlation*[tiab] OR selection*[tiab] OR reduction*[tiab])) OR agreement[tiab] OR precision[tiab] OR imprecision[tiab] OR "precise values"[tiab] OR test–retest[tiab] OR (test[tiab] AND retest[tiab]) OR (reliab*[tiab] AND (test[tiab] OR retest[tiab])) OR stability[tiab] OR interrater[tiab] OR inter-rater[tiab] OR intrarater[tiab] OR intra-rater[tiab] OR intertester[tiab] OR inter-tester[tiab] OR intratester[tiab] OR intra-tester[tiab] OR interobserver[tiab] OR inter-observer[tiab] OR intraobserver[tiab] OR intraobserver[tiab] OR intertechnician[tiab] OR inter-technician[tiab] OR intratechnician[tiab] OR intra-technician[tiab] OR interexaminer[tiab] OR inter-examiner[tiab] OR intraexaminer[tiab] OR intra-examiner[tiab] OR interassay[tiab] OR inter-assay[tiab] OR intraassay[tiab] OR intra-assay[tiab] OR interindividual[tiab] OR inter-individual[tiab] OR intraindividual[tiab] OR intra-individual[tiab] OR interparticipant[tiab] OR inter-participant[tiab] OR intraparticipant[tiab] OR intra-participant[tiab] OR kappa[tiab] OR kappa’s[tiab] OR kappas[tiab] OR repeatab*[tiab] OR ((replicab*[tiab] OR repeated[tiab]) AND (measure[tiab] OR measures[tiab] OR findings[tiab] OR result[tiab] OR results[tiab] OR test[tiab] OR tests[tiab])) OR generaliza*[tiab] OR generalisa*[tiab] OR concordance[tiab] OR (intraclass[tiab] AND correlation*[tiab]) OR error[tiab] OR errors[tiab] OR "individual variability"[tiab] OR (variability[tiab] AND (analysis[tiab] OR values[tiab])) OR (uncertainty[tiab] AND (measurement[tiab] OR measuring[tiab])) OR "standard error of measurement"[tiab] OR ((minimal[tiab] OR minimally[tiab] OR clinical[tiab] OR clinically[tiab]) AND (important[tiab] OR significant[tiab] OR detectable[tiab])AND(change[tiab]OR difference[tiab])) OR (small*[tiab] AND (real[tiab] OR detectable[tiab]) AND (change[tiab] OR difference[tiab])) OR meaningful change [tiab]))

**Exclusion filter** (Terwee et al., 2009)

((((("addresses"[Publication Type] OR "biography"[Publication Type] OR "case reports"[Publication Type] OR "comment"[Publication Type] OR "directory"[Publication Type] OR "editorial"[Publication Type] OR "festschrift"[Publication Type] OR "interview"[Publication Type] OR "lectures"[Publication Type] OR "legal cases"[Publication Type] OR "legislation"[Publication Type] OR "letter"[Publication Type] OR "news"[Publication Type] OR "newspaper article"[Publication Type] OR "patient education handout"[Publication Type] OR "popular works"[Publication Type] OR "congresses"[Publication Type] OR "consensus development conference"[Publication Type] OR "consensus development conference, nih"[Publication Type] OR "practice guideline"[Publication Type]) NOT ("animals"[MeSH Terms] NOT "humans"[MeSH Terms])))))
